# A comprehensive deep learning method for empirical spectral prediction and its quantitative validation of nano-structured dimers

**DOI:** 10.1038/s41598-023-28076-3

**Published:** 2023-01-20

**Authors:** Sneha Verma, Sunny Chugh, Souvik Ghosh, B. M. Azizur Rahman

**Affiliations:** 1 School of Science and Technology, City University of London, London, EC1V 0HB UK; 2 MasterCard, EC4R 3AB London, UK; 3 Department of Electronics and Electrical Engineering, University College London, London, WC1E 7JE UK

**Keywords:** Chemistry, Engineering, Nanoscience and technology, Optics and photonics, Physics

## Abstract

Nanophotonics exploits the best of photonics and nanotechnology which has transformed optics in recent years by allowing subwavelength structures to enhance light-matter interactions. Despite these breakthroughs, design, fabrication, and characterization of such exotic devices have remained through iterative processes which are often computationally costly, memory-intensive, and time-consuming. In contrast, deep learning approaches have recently shown excellent performance as practical computational tools, providing an alternate avenue for speeding up such nanophotonics simulations. This study presents a DNN framework for transmission, reflection, and absorption spectra predictions by grasping the hidden correlation between the independent nanostructure properties and their corresponding optical responses. The proposed DNN framework is shown to require a sufficient amount of training data to achieve an accurate approximation of the optical performance derived from computational models. The fully trained framework can outperform a traditional EM solution using on the COMSOL Multiphysics approach in terms of computational cost by three orders of magnitude. Furthermore, employing deep learning methodologies, the proposed DNN framework makes an effort to optimise design elements that influence the geometrical dimensions of the nanostructure, offering insight into the universal transmission, reflection, and absorption spectra predictions at the nanoscale. This paradigm improves the viability of complicated nanostructure design and analysis, and it has a lot of potential applications involving exotic light-matter interactions between nanostructures and electromagnetic fields. In terms of computational times, the designed algorithm is more than 700 times faster as compared to conventional FEM method (when manual meshing is used). Hence, this approach paves the way for fast yet universal methods for the characterization and analysis of the optical response of nanophotonic systems.

## Introduction

In recent years, many advances in optics have resulted in remarkable capabilities beyond the diffraction limit with various applications in the field of biomedicine, point-of-care applications, and nanotechnology. Nanophotonics has transformed traditional optics by allowing subwavelength structures to influence intense light-matter interactions^1–3^. Nanophotonics strives to use optical resonances and strong surface plasmon localized fields produced by either optimizing their shape or selection of materials.^4–7^ Complex nanostructures, on the other hand, whose shapes may be characterized by several geometrical factors, usually necessitate the use of advanced numerical techniques to deal with multidimensional matrix organizations derived from integral or differential versions of the Maxwell’s equations. There are different numerical techniques available to solve such complex structures based on the finite element methods^8^, such as COMSOL Multiphysics numerical packages^9^, the generalized method of moments (GMM)^10^, and finite difference time domain method^11,12^, as well as the discontinuous Garlekin method^13–15^. Regrettably, simulations employing these tools are often quite time-consuming and computationally costly, nonetheless, in scenarios requiring a real-time application, such as biosensors^16,17^, particle physics^18^, condensed matter^19^, chemical physics^20^, ultra cold science^21^, conventional microscopy^22,23^ iterative inverse designs of complex optical devices^24,25^ and investigation of optical functionalization^26^, efficient modelling of optical performance at the nano/micro region is greatly sought. To overcome this shortcoming, deep learning (DL) techniques^27^ such as multilayer percepteron neural networks (MLPs)^28^, convolutional neural networks (CNNs)^29^, recurrent neural networks (RNNs)^30^, and generative adversarial networks (GANs)^31^, the predictive modelling can play a vital role based on physics has advanced dramatically in the realm of cognitive science^32^. As a result, several researchers have increasingly turned their attentions to DNN and have applied in synchronous transceivers that are one example of these kind of applications^33^, plasmonics^34,35^, multimode fibers^36^, sensing^37–40^, nanotechnology^41–45^ and photonic crystal fibers^46^. Peurifoy et al.^47^ showed the nano shell and they are only predicting scattering cross-section. Baxter et al.^48^showed the plasmonic colours predictions for the sphere and Sajed et al.^49^ is showing the spectra prediction using the convolutional neural networks and recurrent neural networks also the image processing. In this manuscript we focused an array of 3-D structures with evident plasmonic properties. The algorithm predicted the transmission, reflection and absorption spectra that can help to understand the nature of much smaller dimension as their plasmonic properties are quite evident also predicting three spectra which can be the full package to understand the nature of the plasmonic device in terms of resonating wavelength. Hence, in this work tiny coupled nano structures has shown excellent surface plasmon resonance and highly confined electromagnetic fields that may be tuned for diverse purposes by modifying the nano-structures shape and material choices. The overall summary of these findings so far is that a neural network must be developed for a fast computational process and less computing load that involves hyper-parameter tuning, training data production, training of designed neural network, and rigorous testing for each unique problem adopting a specific geometric model.

## Method

### Deep learning neural network (DNN) paradigm and its synchronizations with nanotechnology

**Figure 1:**
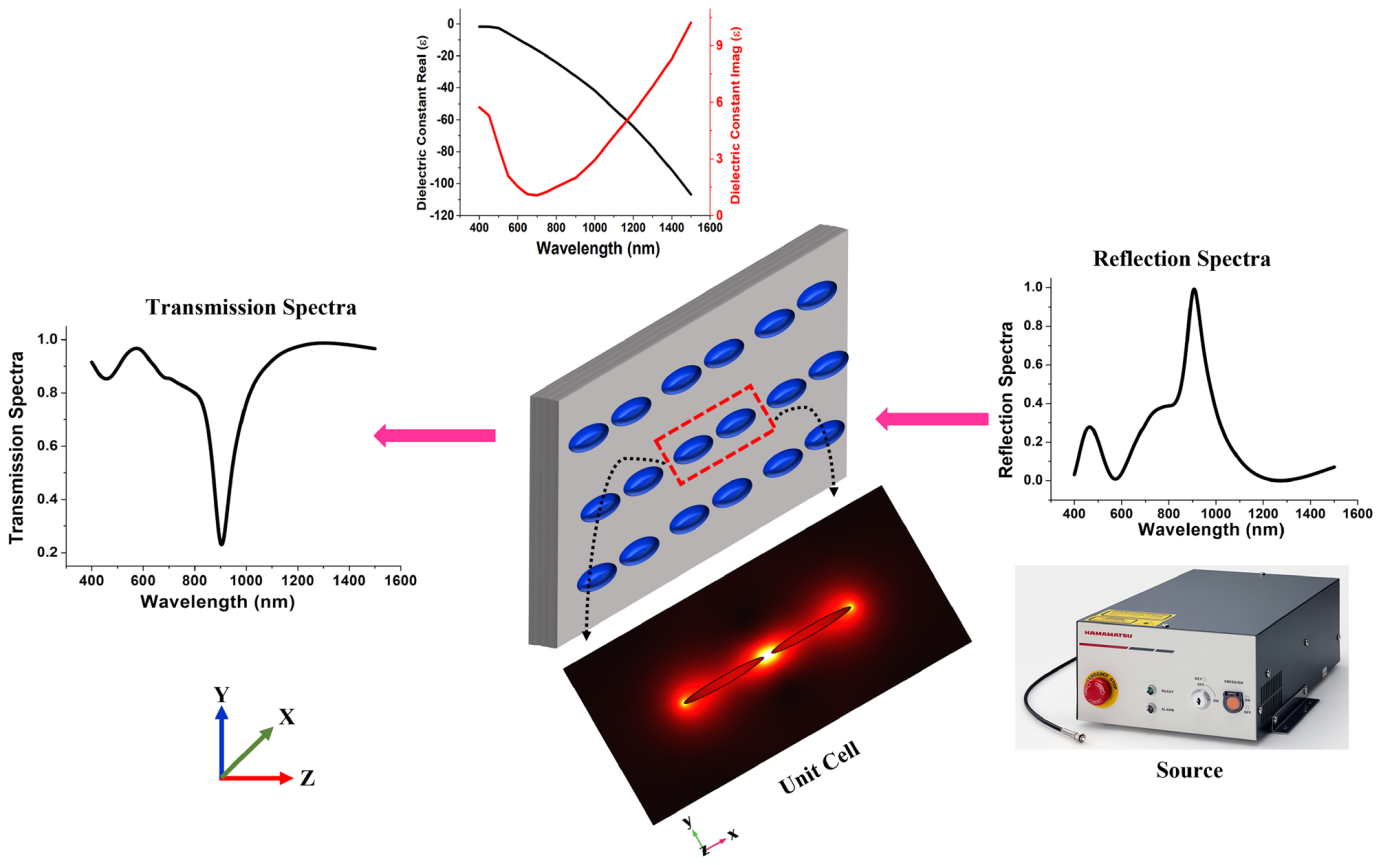
Schematic of the extended unit cell elliptical nano antennas and its optical response in terms of transmitted and reflection spectra.

This work has been organised in two phases where in the first, we have developed a FEM-based frequency domain approach^50–55^ which has been utilized to obtain the surface plasmon resonance confinement around the gold nanostructures. Figure 1 shows an overview of the model description, where gold elliptical and circular dimers have been designed. The dielectric constant of the gold have been adopted from Johnson and Christy^56^. Due to the sheer existence of free electron in the metal, the dielectric constant of the metallic surface was estimated using the Drude free electron model. The dielectric constant of gold is computed with the help of relaxation time $\tau$ = 9.3 ± 0.9 $\times 10^{-15}$ s and for metallic structures which are around near infrared frequencies when $\omega>> 1/\tau$,^56^:

1 $$\begin{aligned} \varepsilon (\omega ) = 1 - \frac{\omega _p{^{2}}}{\omega ^2} +j\frac{\omega _p{^{2}}}{\omega ^3\tau } = \varepsilon {}^f_{real}+ j\varepsilon {}^f_{imag} \end{aligned}$$

Here the plasma angular frequency is defined by $\omega _p{}$ which is equal to $\sqrt{\frac{4 \pi Ne^2}{m_0}}$ = 9 eV, where $m_0 = 0.99\pm 0.04$ defined as effective optical mass and *N* is the conduction electron density^56^.

The classical Maxwell equations were solved by employing the FEM, taking into account the harmonic dependency of the electric field $\textbf{E}(r,t) = \textbf{E}(r)e^{-jwt}$ in order to examine the physical plasmonic characteristics of nanostructures. Throughout the simulations, the Helmholtz equation has been employed, which can be obtained from the usual Maxwell equations, as shown below.

2 $$\begin{aligned} \nabla ^2 E +k_0^2 \varepsilon E = 0 \end{aligned}$$

Here $k_0$ is the wave vector. Temporal periodic distributed field was used as $E(x,y,z) = E(x,y,z)e^{j\beta z}$, and $\beta$ is defined as a propagation constant. In complex form, $\gamma = \alpha + j\beta$, and if $\alpha = 0$, then $\gamma = j\beta$, which represents the loss-less propagation dependency in the *z*-direction. To excite the nanostructures, the x-polarised wave is launched from the top layer in the z-direction which generated the LSPR upon interaction with the designed nanostructures. The strongly localized LSPRs and its optical responses for elliptical has also been shown in the inset of Fig. 1. For more details please see Sect. I of the Supporting Information. From supplementary information, it is clearly proved that the frequency response is sensitive to geometrical parameters of any nanostructure, materials characteristics, and changes in the local environment, LSPRs have a huge potential for molecular sensing, which could help in clinical diagnosis, environmental monitoring, and detection of biological agents^57–59^.

## Results

**Figure 2:**
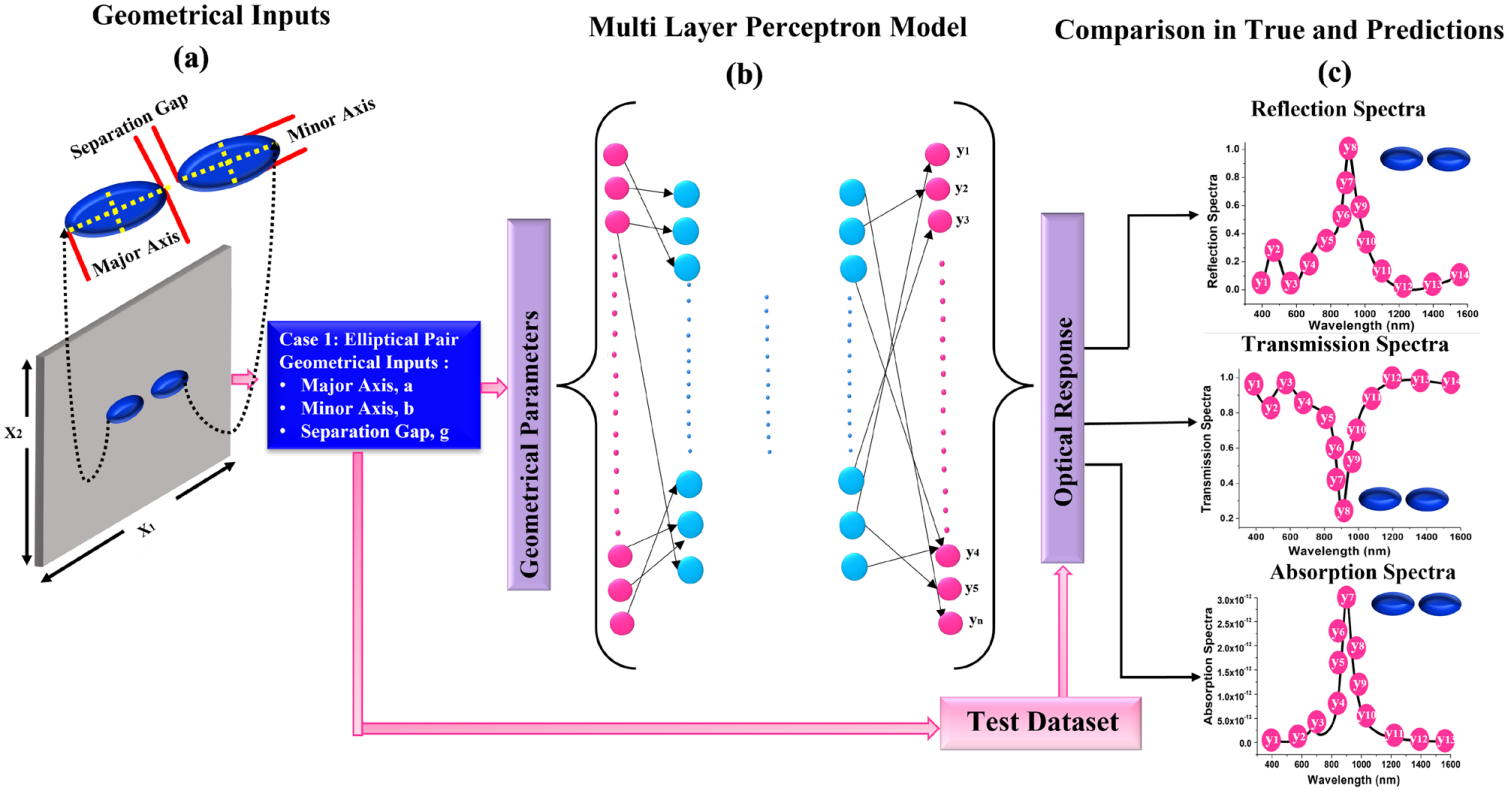
(**a**) Schematic of the structure of two elliptical nanostructures on $SiO_{2}$ substrate (The top and front views of a single unit cell with the geometrical parameters are represented in the insets). (**b**) A DNN model that predicts the optical response over a wavelength range for the given geometrical parameters and (**c**) Shows the predicted optical Reflection, Transmission and Absorption responses for a given geometrical structure.

The analyte molecules are typically attached to the exterior face of the nanostructures, either along with or without tethering particles. It generates a small perturbation of the dielectric surrounding refractive index (RI), resulting in a measurable shift in the resonance frequencies or amplitude, which may be evaluated instantaneously using the transmission, reflectance and absorption spectra which can be predicted with the help of designed DNN configuration as shown in Fig. 2.

Here Fig. 2a shows the given geometrical inputs (paired elliptical) to the DNN. Surface lattice resonances (SLRs) are made up of gold nanostructures organised in a regular pattern. It can sustain resonances that are formed via LSPRs coupling and have much finer spectral characteristics^60^. A gold nanostructure on a Si substrate supports plasmonic resonances in each unit cell of the structure. The geometric properties of the nanoparticles^61^, which may be mapped to the major (*a*) and minor (*b*) axes for elliptical dimer, sepration distance (*g*) and height (*h*) of the nanostructures, influence the wavelengths at which SLRs are triggered. Variation in these parameters can change the optical spectral characterstics. Thus, the major (*a*) and minor (*b*) axes, separation gap (*g*) are adopted as a input parameters, and the corresponding outputs are discrete spectral datapoints in the Visible-Infrared region . Figure 2b shows the architecture of the developed neural network when the input parameters are used for predicting the spectral response of the corresponding nano structures. At the start of training of the developed DNN, the learning algorithm develops an estimated function that predicts output values. After adequate training, this built model is expected to produce output spectral responses for any new input geometrical dimensional value. This process of learning will determine the mean squared errors (*MSE*) to demonstrate the efficacy of the proposed DNN by comparing its anticipated spectral output with the actual spectral values. Several widely used machine learning packages were evaluated to develop and train this DNN, including *pandas*^62^ for data preprocessing and *Scikit-learn*^63^ for intensive training. *NumPy*^64^ for matrices and multidimensional arrays, and *pickel*^65^ for compiling and deserializing a *Python* object hierarchy are all elevated libraries used for developing regression model. Subsequently, *Pytorch*^66^, a DNN framework centred on Torch tensors, was employed. It’s a free licence package created in AI Research lab organized by Facebook (FAIR)^67^ in 2016 and entrenched on the scripting language *Lua*^68^, that is identical to *NumPy* with GPU integration. This is a crucial method since it assists in the acceleration of numerical computations, which may strengthen the performance of the DNN upto 60 times. It has a more concise and easier to read Application Programming Interface (API), making it simpler to integrate with *Python*. The usage of this excellent platform is attributable to the fact that it facilitates the creation of rapid computational features that can be updated in real-time, which is necessary throughout DNN training process. Designers used FEM solvers in the back-end for dataset collection, which is useful to train the DNN, and *Pytorch* and *Scikit-learn* in the front-end due to their remarkable compelling architectural style, which facilitates rapid and lanky approaches, even though *PyTorch* employs several backend instead of a single backend for GPUs and CPUs as well as other operational aspects. While designing this algorithm, *Adam Optimizer* has been used in this work because it is widely assumed that Adam converges faster than vanilla Stochastic Gradient Descent (SGD) and Stochastic Gradient Descent (SGD) with momentum.^69^. Due to this reason, we have selected *Adam Optimizer* as it works best for the nonlinear datasets and it also has the capability to update the learning rate for each parametric values because it adapts first-order gradients with a minimum memory requirements^70^. The weights and bias values of the designed DNN are optimized and updated iteratively by minimizing MSE using with the help of *Adam*^71^. Hence, the designed algorithm is suitable to analyse/predict/discern the optical response of the paired nanostructures.

### Architectural framework of DNN with empirical attestation

DNN have indeed been established as a powerful tool for deciphering the correlation between the architecture and re-configurable nanophotonic structure composition and its functionality. It involves the construction of computer algorithms that aid in the extraction of motifs and the optimization of complicated information with a large number of variables. Forward ANNs are remarkable in that they may leverage numerous layers and *neurons* to operate efficiently. This neural network is formed using a cognitive computer with 8 GB RAM, 500 GB Hardrive, with the windows operating system (version 20H2 Semi-Annual Channel) installed. Throughout the calculation, the virtual environment Spyder python (version 5.1.5) is installed in anaconda (version 1.7.2). This DNN was arranged in three levels, as shown in Fig. 2b, including an input, output and *hidden layers*. The input parameters that must be interpreted are delivered to the fully linked input layers. Prediction and categorization are among the tasks that the output layer performs. A layer-by-layer assembling of *neurons* makes up a neural network. Every *neuron* in single layer is interconnected to the *neurons* in the following layers via a weighted connection. The frequency of the relation between the $j_{th}$ *neuron* in one layer and $i_{th}$ *neuron* in other is represented by the weight $w_{ij}$. Each *neuron* is given a function weight, which is then linearly aggregated (or summed) and transmitted with the help of an activation function to produce the output from *neurons*. Finally, the anticipated output data may be compared to the random test data points. The designed DNN can be visualised as a closed box that accepts *x* inputs and generates *y* outputs^72^ (see Fig. 2b). As shown in Fig. 2, an optimal DNN with optimized *hidden layers* = 5, *neurons* = 50 in each layer was implemented throughout this investigation. Every *neuron* inside each layer was interconnected to the *neurons* in the subsequent layer, implying that these concealed levels were totally integrated. 20% of datapoints were randomly adopted from the training datapoints and supplied as the evaluation datapoints to provide impartial evaluation while tweaking the DNN hyperparameters (weights and biases).

## Discussion

**Figure 3:**
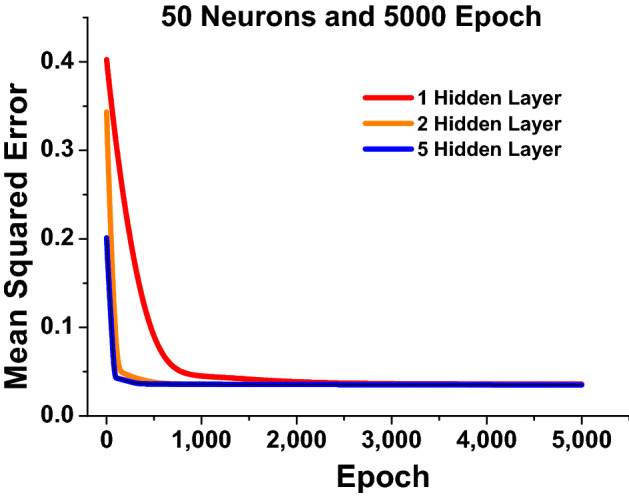
Shows the *MSEs* values (Validation Set) for 1, 3 and 5 *hidden layers* when *neurons* = 50 and *epoch* = 5000.

In this work, the geometrical parameters (*a*, *b*, *d* and *g*) of the nanostructure were varied from 10 nm to 130 nm; however, in this work for simplicity *h* was fixed at 40 nm. The granularity of gathered dataset is chosen to minimise computing costs while yet allowing the DNN to be trained properly. The complete datasets throughout this investigation comprise 10,500 parameter combinations and their accompanying spectra. We exclusively selected structural factors that have a considerable influence on the spectral properties and cover all conceivable spectrum variants. Indeed, with this selected quantity of training data, DNN can be trained to accurately model and forecast millions of spectral properties of the plasmonic structures in the parametric range. Datasets are divided into three groups throughout the training process, training dataset, validation dataset, and test dataset. More details about the dataset preparation are presented in the Sect. II of supporting information. Training dataset are provided to the DNN to optimise the algorithm by revising weights whereas validation dataset are used to evaluate the DNN, acting as a verification of the training response and supporting to determine if the network is overfitting; and test dataset are used to assess the predictive performance. Each time, the ideal DNN is determined by selecting suitable hyperparameters depending on the training performance. The performance improvement of the DNN are thoroughly investigated in terms of *MSEs* that have been calculated for each *hidden layers* when the *epoch* = 5000 and *neurons* = 50. For *MSEs* calculation following Eq.(3) has been used.

3 $$\begin{aligned} MSEs = \frac{1}{n}\sum _{i = 1}^{n}\left( Z_{i}^{a} - Z_{i}^{p}\right) ^2 \end{aligned}$$

where n is the total number of datasets utilised throughout the training process. $Z_{i}^{a}$ is the original data points calculated using COMSOL multiphysics, and $Z_{i}^{p}$ is the predictions over the actual dataset. The calculated *MSEs* of the predicted datapoints from the developed network compared to the targeted datapoints are quantified by *MSEs*, which itself is regarded the most essential effectiveness assessment criterion. It is also used as the validation criteria of the DNN. Hence, the comparison of the *MSEs* calculation at each *hidden layers* are shown in Fig. 3 when *neurons* = 50 and *epoch* = 5000.

Figure 3 shows that the variation of *MSE* values with epoch for *hidden layers* = 1, 3 and 5 when *neurons* = 50 and *epoch* = 5000. Here, the red curve shows the *MSEs* values of 0.4 at *epoch* = 1 for *hidden layers* = 1, which rapidly decreases until *epoch* = 2000, and then almost constant for *epoch* $\ge$ 2000. The orange curve depicts the *MSEs* values of 0.35 at *epoch* = 1 but for *hidden layers* = 2, and it rapidly stabilized after *epoch* $\ge$ 500. Sect. II of Supplementary Information contains additional information on the remaining estimated *MSEs*. On the other hand, at *epoch* = 1, *MSEs* values of 0.2 is shown by a blue curve when the *hidden layers* = 5, *neurons* = 50, respectively. The *MSEs* values fall significantly faster for *hidden layers* = 5, and in this context, it can be stated that the constructed neural network produces a better approximation when the hyper-parameters are adequately configured.

**Figure 4:**
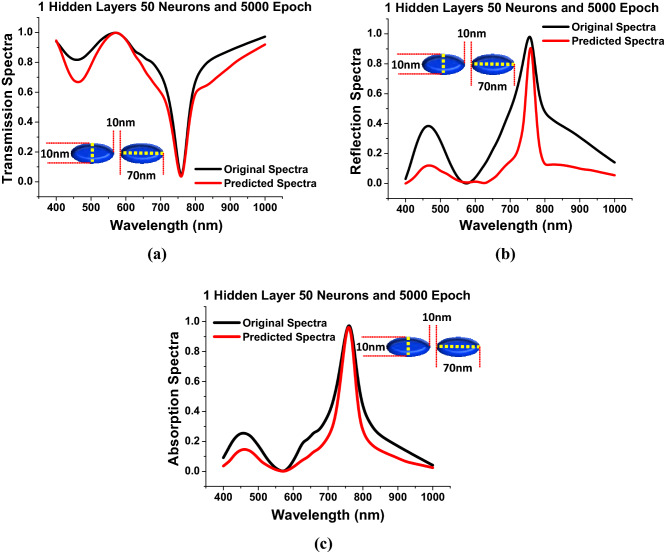
*Hidden layer* = 1, *Neurons* = 50 and *Epoch* = 5000 (**a**) Shows the comparison between the predicted transmission spectra with respect to the original transmission spectra. (**b**) Shows the anticipated reflection spectra are compared to the original reflection spectra. (**c**) Shows the predicted absorption spectra against the original absorption spectra.

For selecting the best hyper-parameters in terms of performance of the DNN, the *hidden layers* are optimized in first stage when the number of *epoch* and *neurons* were fixed at 5000 and 50. The initial prediction have been made for the given input geometrical dimensions as *a* = 70 nm, *b* = 10 nm, and *g* = 10 nm and the corresponding predicted transmission, reflection and absorption spectra shown in Fig. 4 when *hidden layers* = 1.

In Fig. 4a the black curve shows the original transmission spectra (calculated by COMSOL Multiphysics) along with the predicted transmission spectra shown by the red curve, when the *a* = 70 nm, *b* = 10 nm, *g* = 10 nm and *h* = 40 nm. Similarly, the predicted reflection and absorption spectra are also shown in Fig. 4b,c, respectively, where the original spectral values are shown by the black curves, while the predicted values are represented by the red curves. Here, it can be observed (shown in the supplementary materials) that when *hidden layers* = 1 and *neurons* = 50, the *MSEs* was calculated as 0.4 for *epoch* = 1 and rapidly reduces till *epoch* = 900; however, it got stabilised after *epoch* = 1000. Hence, so far *epoch* = 5000 is used to make initial predictions. Indeed, it is true that at a lower *MSEs*, the number of predicted spectral values are closer to their actual values. Due to this reason, the remaining hyper-parameters have been tweaked for producing more accurate predictions over the actual spectral responses. More information on hyper-parameter tweaking can be found in Sect. II of the supporting material.

In Fig. 3 it was shown, as the number of hidden layers is increased, the predicted results became better. Finally, the appropriate DNN framework is designed using suitable hyper-parameter selection based on the *MSEs* calculated at every dataset training. In the Final algorithm the *hidden layers* = 5, *epoch* = 5000 and *neurons* = 50 were adopted. The *MSE* had its minimum values 0.20 at *epoch* = 1 and reduces upto 0.05 at *epoch* = 200; however, it stabilises and reaches nearly to 0 at *epoch* = 5000.

**Figure 5:**
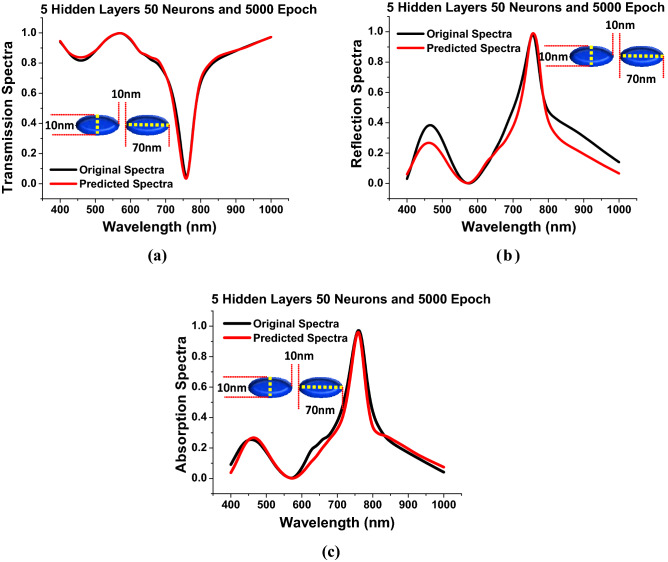
*Hidden layer* = 5, *Neurons* = 50 and *Epoch* = 5000 (**a**) Shows the comparison between the predicted transmission spectra with respect to the original transmission spectra. (**b**) Shows the anticipated reflection spectra are compared to the original reflection spectra. (**c**) Shows the predicted absorption spectra against the original absorption spectra.

Figure 5 illustrates that as the hidden layer is increased to 5, the outcomes form the improved DNN can be clearly seen that as the *MSEs* is reduced the predicted transmission, reflection and absorption responses reaches closer to the original spectral values shown by red and black curves, respectively for the specified geometrical dimensions taken as *a* = 70 nm, *b* = 10 nm, *g* = 10 nm and *h* = 40 nm. Altogether, the findings suggest that DNN can accurately predict spectra for billions of distinct nanostructures in the *a*, *b*, *g* and *h* ranges using adequate amount of simulation dataset. They all predict the same accurate resonance properties as by FEM simulations (using COMSOL Multiphysics), demonstrating that the DNN can be well trained for electromagnetic modelling. As a result, it is reasonable to conclude that expanding the training dataset will improve the performance and accuracy of the DNN.

**Figure 6:**
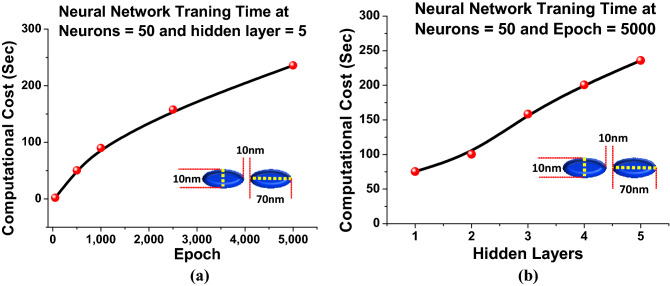
(**a**) Shows the computational Cost (Sec) for the DNN training with respect to different *epoch* (**b**) Represents the comparison in computational cost for different *hidden layers*.

The performance of the designed neural network has also been evaluated in terms of the computational cost. Generating large training data sets for DNN demands a significant investment of computational effort. This emphasises the critical difficulty of automatically generating extra data points, particularly for regions that are not included in the present data collection. Aside from reducing numerical efforts, this would also aid to cut physical labour by reducing the involvement of the researchers in the data curation chain. However, the high computational cost of producing such data sets typically hinders database expansion; as a result, the resulting DNN can be unreliable owing to over-fitting and other difficulties. Hence, Fig. 6a depicts the comparison of training execution time times for different *epoch* when *neurons* = 50 and *hidden layers* = 5. Here, computational cost for 10,500 training data points have been calculated while DNN trains. It is evident that the developed DNN have shown approximately 1 sec at *epoch* = 1. However, it takes up to 50 s for *epoch* = 500 and for *epoch* = 5000 takes 236 s. In Sect. II of the supplementary material it is shown that at lower *epoch* the *MSEs* values were high and the predictions were not closer of the actual results.

Hence, computational cost has been compared at different *epoch*. It can be stated that at every *epoch* weights and parameters were stored in the computing machine after the DNN training was finished and the predictions were made for unseen inputs with the aid of previously saved weights at *epoch* = 5000 is also represented in Fig. 6. With the increment of number of epoch the computational cost increases whereas the cost per epoch reduces. As a consequence, it can be inferred that at *epoch* = 5000, although the computational cost is 236 seconds, which is rather expensive when compared to the smaller *epochs*, but the performance of the DNN is improved. This performance is also far superior to typical FEM solvers, which may take up to 8100 s, 10,200 s, 14,100 s, 38,160 s, 86,400 s and 17,2800 s to compute the optical spectrum responses of a single dimer using coarse, normal, fine, finer, and extremely fine and manual meshes. We cannot avoid the effort and computational cost that has been utilised to collect the vast amount of the dataset by using EM solvers. However, it is an one time process. Once the model is fully trained, it can quickly predict the solutions for any unseen values compared to traditional EM solvers. Next, Fig. 6b also shows the computational cost of the DNN when the *hidden layers* was increased from 1 to 5. Here, it can be seen that at *hidden layer* = 1 the computational load was comparatively small, approximately 75 s but in Fig. 4 It was shown that the spectral performances was not acceptable hence the DNN training has been continued for a larger number of *hidden layers*. It can be seen that at *hidden layers* = 2, 3, 4 and 5 the computational cost increases to 100 s, 170 s, 220 s and 236 s, respectively when a fixed 5000 epoch was used. However, it should be noted as shown in Fig. 3, for a higher hidden layer, a smaller epoch can be satisfactory. Additionally, the corresponding improvement in *MSEs* values were also presented in Fig. 3 from where it is clear that as the *hidden layers* is increased the *MSEs* values are decreased which suggests the prediction are getting more closer to the actual spectral values. Hence, the *epochs* = 5000 is selected by the user once *MSEs* has converged to a suitable threshold. After modifying the model to obtain a stable MSE value, the necessary outputs datapoints were provided as additional input datapoints that was not supplied during the training operation.

**Figure 7:**
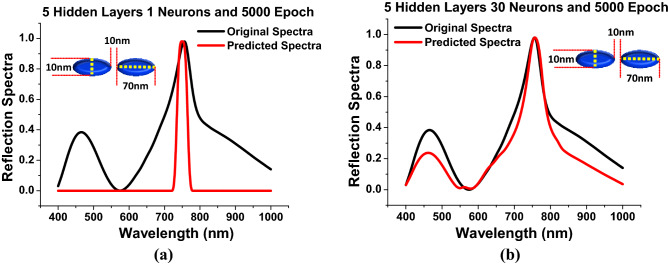
*Hidden layers*= 5 and *Epoch* = 5000 (**a**) Shows the comparison of predicted reflection spectra with respect to the original reflection spectra at *neuron* = 1 (**b**) Represents the resemblance of predicted reflection spectra with respect to the original reflection spectra at *neuron* = 30

Next, the effect of the number of *neurons* for a fixed *hidden layers* = 5 and fixed *epoch* = 5000 is studied and shown in Fig. 7. The *neuron* assesses a set of weighted inputs, implements an activation function, and obtains the outputs. An input from *neuron* might be either features from a training set or outputs from *neurons* in a previous layer. Weights are assigned to inputs as they travel through synapses on their route to the neuron. The *neuron* then applies an activation function (ReLU in this case) to the ”aggregate of synaptic weights” from each arriving synapse and sends the result to *neurons* of following layer. Hence, ReLU implementation is the most significant breakthrough milestones in this network. This can be better followed from Fig. 7a which shows the performance of the predicted spectra when the *hidden layer* = 5, *epoch* = 5000 and *neurons* = 1. From this it can be seen that although the number of *hidden layer* and *epoch* were sufficient still the numbers of *neurons* also plays an important role for stabilizing the DNN. Hence, the predicted spectral values at *neuron* = 1 also plotted where red curve shows the predicted spectral values is very different than the actual results shown by a black curve for *a* = 70 nm, *b* = 10 nm, *g* = 10 nm and *h* = 40 nm. However, as the number of *neurons* is increased to 30, Fig. 7b shows the response with *hidden layers* = 5 and *epoch* = 5000. From this it can be inferred that as the *neurons* increases the performance of the predicted spectral values significantly improves where the red curve shows the predicted spectral response with respect to original spectral values shown by a black curve. Hence, it can be stated that as the number of *neurons* increases the algorithm converges and reaches at it saturation point so the outcomes of the final optimized hyper-parameters when *hidden layers* = 5 and *epoch* = 5000. To show convergence in the predicted spectral values more clearly, the *MSEs* has been plotted at different number of *neurons* when the *epoch* varies from 0 to 5000 and *hidden layer* = 5. As, it has been already discussed that at the lower *MSEs* the prediction will be more accurate towards the original spectral values.

**Figure 8:**
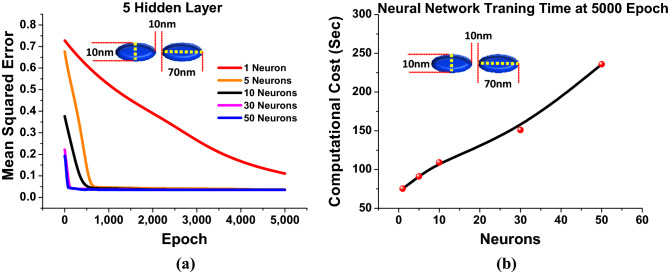
*Hidden layer* = 5 and *Epoch* = 5000 (**a**) Shows the comparison of *MSEs* at different *neurons* (**b**) Represents the trend of the computational cost with respect to the variations in *neurons*

Figure 8a shown the improvement in *MSEs* when *neurons* number increased from 1 to 50. Here, the red curve shows the highest *MSEs* values = 0.75 at *epoch* = 1 and gradually reduces till 0.1 at *epoch* = 5000 which suggest that the prediction with *neuron* = 1 is not acceptable to make an efficient DNN as seen in Fig. 7a. Hence, to improve the performance 5 *neurons* has been used and the corresponding *MSEs* are shown by a orange curve. In this case, the *MSEs* values = 0.68 at *epoch* = 1 and sharply reduces till *epoch* = 1000. Finally, it stabilised till *epoch* = 5000 when *neuron* = 10 are used, as shown in Fig. 8a. For *neuron* = 10, shown by a black curve shows the expeditious reduction in *MSEs* values = 0.38 at *epoch* = 1 and decreases till *epoch* = 700 until stabilised. For *neuron* = 30, pink curve shows the further decrements in *MSEs* values = 0.22 at *neurons* = 30 and steadies after *epoch* = 200. The corresponding spectral response is also shown in the Fig. 7b. Finally, for *neurons* = 50 shown by a blue curve have been adopted to see further improvement in the *MSEs* values, when the *MSEs* values = 0.18 at *epoch* = 1 and quickly stabilised after *epoch* = 50. Hence, *neurons* = 50 was considered for further observations as it is showing lower *MSEs* values and faster convergence. The corresponding predicted spectral values are also shown in Fig. 5. Figure 8b shows the effect of computational cost at different *neurons* when *hidden layer* = 5 and all of them with *epoch* = 5000. Here, it is clear that when number of *neurons* = 1 the computational time was 50 sec; however, it increased more and reaches upto 250 sec for *neurons* = 10 but the DNN stabilised. However, it should be noted that when the larger number of neurons is used, the number of epoch can be reduced. This computational cost also depends on the specifications on the computing machine.

## Substantiation of in-house developed DNN for concealed nanostructures

**Figure 9:**
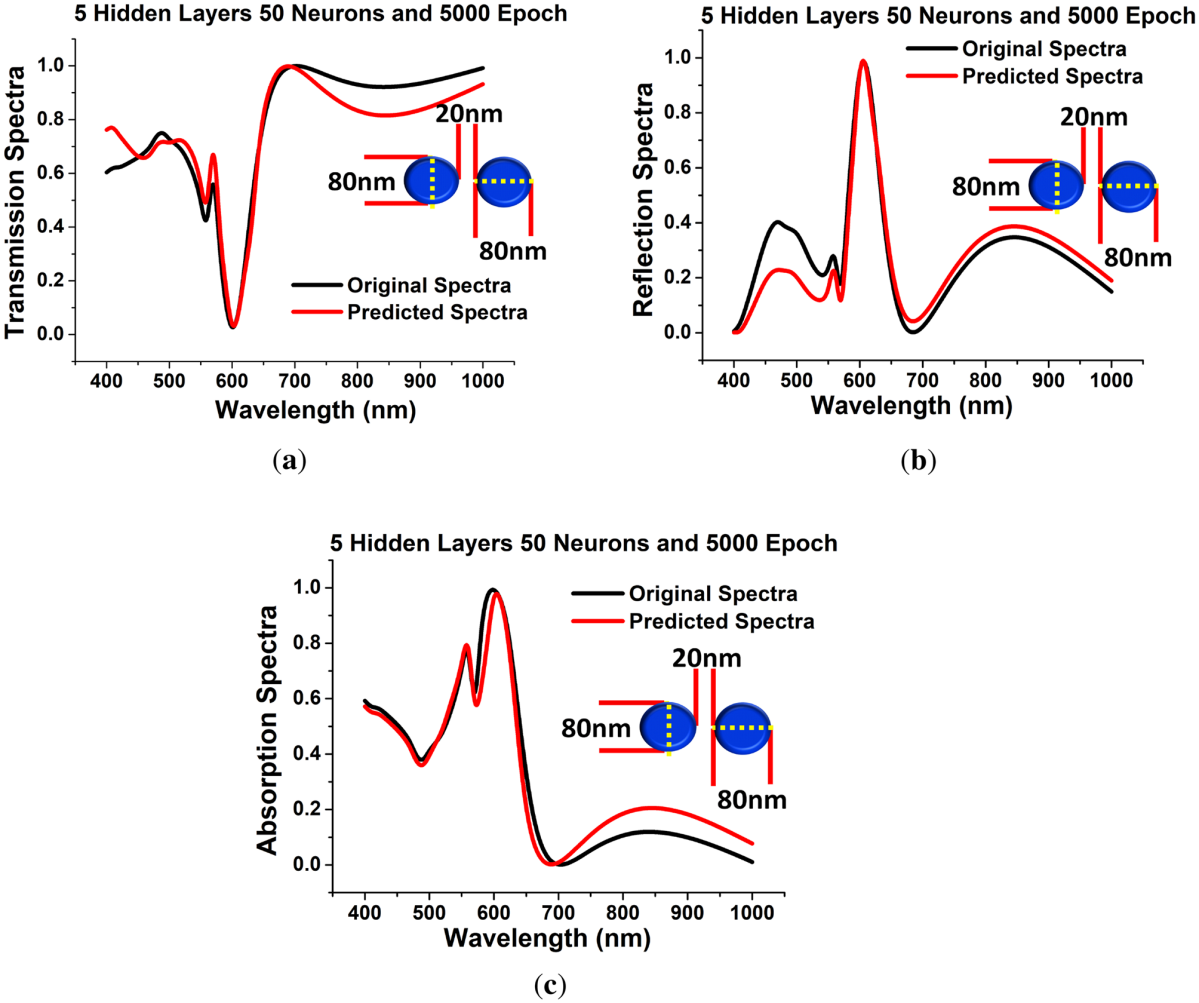
*Hidden layer* = 5, *Neurons* = 50 and *Epoch* = 5000 (**a**) Shows the comparison between the predicted transmission spectra with respect to the original transmission spectra of circular dimer with *d* = 80 nm, *g* = 20 nm and *h* = 40 nm. (**b**) Shows the anticipated reflection spectra are compared to the original reflection spectra. (**c**) Shows the predicted absorption spectra against the original absorption spectra of the same.

Finally, after stabilizing the developed DNN with the help of the all possible hyper-parameters, we have demonstrated in this paper how the deep learning and dynamic challenges are interconnected, providing the groundwork for future research at the intersection of problems and data science. In particular, we suggest novel topologies for DNN that increase forward propagation stability. Using the derivative-based learning regularisation the well-posedness of the learning activity was increased. Moreover, presented a multi-level technique for establishing hyper-parameters, which makes DNN training easier. Further introduced new regularisation techniques that rely on our continuous conceptualization of the challenge to increase generalisation accuracy, consistency, and streamline DNN training. After designing a stable DNN, we have used this algorithm for predicting the spectral response for the paired *circular* nano structure where *d* = 80 nm, *g* = 20 nm and *h* = 40 nm. Figure 9a shows the spectral response of a paired *circular* nano disk where a red curve shows the predicted spectral values and their actual spectral values calculated by FEM are shown by a black curve. Similarly, Fig. 9b,c show the predicted reflection and absorption spectra (shown by red curves) and actual reflection and absorption values are shown by black curves. These results show, when *hidden layers* = 5, *neurons* = 50 and *epoch* = 5000 are used to predict the transmission, reflection and absorption spectra then these are close to the actual spectra.

### Evaluation of in-house developed DNN for imperceptible geometric dimensions (beyond the training dataset)

**Figure 10:**
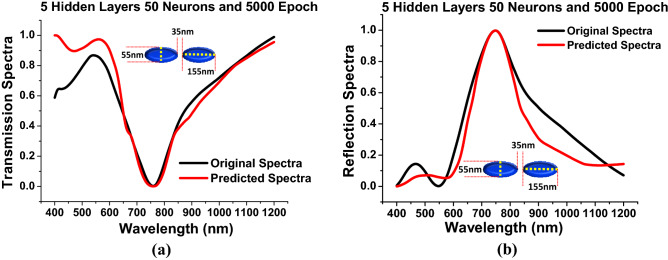
*Hidden layer* = 5, *Neurons* = 50 and *Epoch* = 5000 (**a**) Shows the comparison between the predicted transmission spectra against the original transmission spectra. (**b**) Shows the anticipated reflection spectra are compared to the original reflection spectra when *a* = 155 nm, *b* = 55 nm, *g* = 35 nm and *h* = 40 nm.

In this section, we have discussed the performance of the designed DNN when it predicts the spectral values outside the range of the training dataset. The geometric parameters are selected at random from the test sets, but outside of the training dataset and verified by using the commercial software for the plasmonic nanostructures to examine the performance optimization of the transmission and reflection values for an arbitrary wavelength and visualize the outcomes. During the entire training period, we have used the dataset of major axis (*a*) from 10 to 130 nm with 10 nm interval. Hence, in this section the spectra has been predicted when major axis (*a*) = 155 nm, minor axis (*b*) = 55 nm and separation gap (*g*) = 35 nm, and it should be noted that these values were not available in the training set. It is worth to note that Fig. 10 shows spectral response of the optimised DNN with prediction accuracy and reliability more than 90$\%$ when approximately 50,000 dataset were used for training to show the impact of the test set, which was outside the range of training data set. Here, black curve shows the original spectral values computed by using COMSOL multiphysics and the red curves shows the spectral values predicted from the in-house developed neural network.

A significant facilitator of cutting-edge nanotechnology research would be the capability to swiftly extract a required optical response by using artificial neural network from the geometrical parameters of a plasmonic nanostructures. One can envision a variety of scenarios in which such data is essential to the design investigations of any nano structures. The highlight of this DNN is that it has a capacity to address multiple targeted resonance spectra for various paired geometrical dimensions, and it emphasises that this technique may be applied to other sensing in biology, chemistry, and material science. Hence, it can be said that the spectrum prediction from the nanostrutural recognition have a high degree of employability, indicating that this techniques might indeed be useful in a wide range of spectral and non-spectral aspects. This deep learning protocol has the potential to revolutionize real-time field applications in a variety of spectroscopic disciplines.

## Conclusion

In conclusion, this work demonstrates the use deep learning to correlate spectroscopic knowledge of a paired nanostructure in local environments. The presented DNN algorithms can estimate spectral values of designed paired nano structures at more than 700 times lower computing cost than the traditional FEM solver (when manual meshing is used) while providing the similar degree of precision. This study illustrates DNN has been tested rigorously and shown its excellent predictions using one time trained process. *Hidden layers* = 5, *neurons* = 50 and *epoch* = 5000 were employed all across the neural network to provide a swift convergence and yet good precision in estimating spectral values for randomized input geometrical dimensions of the paired nanostructures. These values can depend on the type of the problem. However, as the results may not be known beforehand so for a real application a safer number of these DNN parameter can be used. In this work, we have also shown the performance of the associated hyper-parameters of the designed DNN and explained in terms of *MSEs* which is plotted with respect to *hidden layers*, *epoch* and *neurons*. This research also offers a contrast between traditional FEM solver and in-house developed DNN in terms of computing time, which is more than 700 times faster than direct FEM simulations (when manual mesh size is used). Finally, the performance of the proposed DNN model was proven for the random input parameter for inside and outside the training dataset such as paired circular when *d* = 70 nm and *g* = 20 nm and paired elliptical dimers when *a* = 155 nm, *b* = 55 nm and *g* = 35 nm respectively, and corresponding spectral values were correctly predicted. The detection of structural variations/fluctuations in chemical reactions, automatic identification of interstellar molecules, and real-time recognition of particles in biomedical diagnosis are just a few application when deep learning can be exploited. Thus, we conclude that the consolidation of nanotechnology and artificial intelligence will open the direction for many other new technological advancements in the profession of comprehensive scientific disciplines.

## Supplementary Information

Supplementary Information.

## Data Availability

All data generated or analysed during this study are included in the supplementary information in the graphical form. The raw datasets and computational models used and/or analysed during the current study available from the corresponding author on reasonable request.
