# Supplementary material for: A comprehensive deep learning method for empirical spectral prediction and its quantitative validation of nano-structured dimers
Source: Sci Rep. 2023 Jan 20;13:1129. doi: 10.1038/s41598-023-28076-3 (PMC9860028; doi:10.1038/s41598-023-28076-3)
Supplement: Supplementary file 1 — Supplementary Information. [file 41598_2023_28076_MOESM1_ESM.pdf]

Section I: FEM Calculations

Electromagnetic Theory

To calculate the electronic transition of the metal Drude Lorentz model has been used because it focuses on the free-electron present in the metals which causes surface plasmons resonance. Drude Free electron model is proven to be more suitable for the materials which have more free electrons as compared to bound electrons. Dielectric constant was calculated according to the Drude's free-electron theory[1] by using Eq. S.1:

$$\varepsilon(\omega) = 1 - \frac{{\omega_p}^2}{\omega(\omega + j/\tau)} \tag{S.1}$$

Here, ω_p is the plasma angular frequency equal to $\sqrt{\frac{4\pi Ne^2}{m_0}}$, where N and m_0 are the conduction electron density and effective optical mass respectively, and τ is the relaxation time. This can be separated into its real and imaginary parts of the dielectric constant[1].

$$\varepsilon_{real} = 1 - \frac{{\omega_p}^2 \tau^2}{1 + \omega^2 \tau^2} \tag{S.2}$$

$$\varepsilon_{imag} = \frac{{\omega_p}^2 \tau}{\omega(1 + \omega^2 \tau^2)} \tag{S.3}$$

For metals at near-infrared frequencies when $\omega >> 1/\tau$, Eqs. (S.2 and S.3) simplified with the help of Johnson and Cristy[1].

$$\varepsilon(\omega) = 1 - \frac{{\omega_p}^2}{\omega^2} + j\frac{{\omega_p}^2}{\omega^3 \tau} = \varepsilon^f_{real} + j\varepsilon^f_{imag} \tag{S.4}$$

The calculated real and imaginary parts of the dielectric constant are shown in Figure 1, which have been used to calculate the transmission and reflection coefficients.

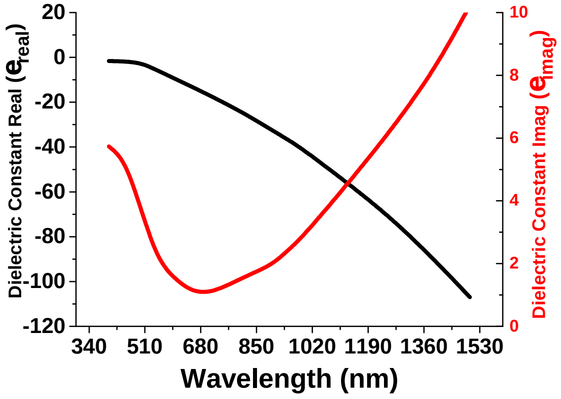

Figure 1. Real and Imaginary parts of the dielectric constant of gold data from Johnson and Cristy[1]

In order to investigate the physical plasmonic properties, conventional Maxwell equation is solved by using the FEM considering the harmonic dependence of Electric field $E(r,t) = E(r)e^{-jwt}$. Helmholtz equation can be derived from the Maxwell's equations and shown below,

$$\nabla^2 E + k_0^2 \varepsilon E = 0 \tag{S.5}$$

where k_0 is the wavevector and the time harmonic the propagating field is defined as $E(x,y,z) = E(x,y,z)e^{j\beta z}$ and where β is the propagation constant. In complex form $\gamma = \alpha + j\beta$, if $\alpha = 0$ then $\gamma = j\beta$ represents the propagation dependence in the z direction. A x-polarized wave in z-direction is launched from the top surface to excite the antenna, which generates the LSPR after the interaction with the proposed gold nano shapes.

Computational model Description:

The schematic diagram of a paired gold nano structured antenna on the quartz substrate is shown in Figure 2, the unit cell of quartz substrate on which a paired gold nano structure is placed. All the numerical simulations were carried out by using COMSOL Multiphysics based on the FEM. To reduce the simulation time, we have simulated one unit cell enforcing periodic boundary conditions as shown in Figure 2a. The metal disk was excited by x-polarized light launched in the z-direction from the top with wave excitation 'ON' and additionally Scattering boundary conditions (SBC) has been placed at the bottom and top of the computational domain. The entire numerical problem was solved in frequency domain to obtain the scattered field distributions and the transmission and reflection spectra.

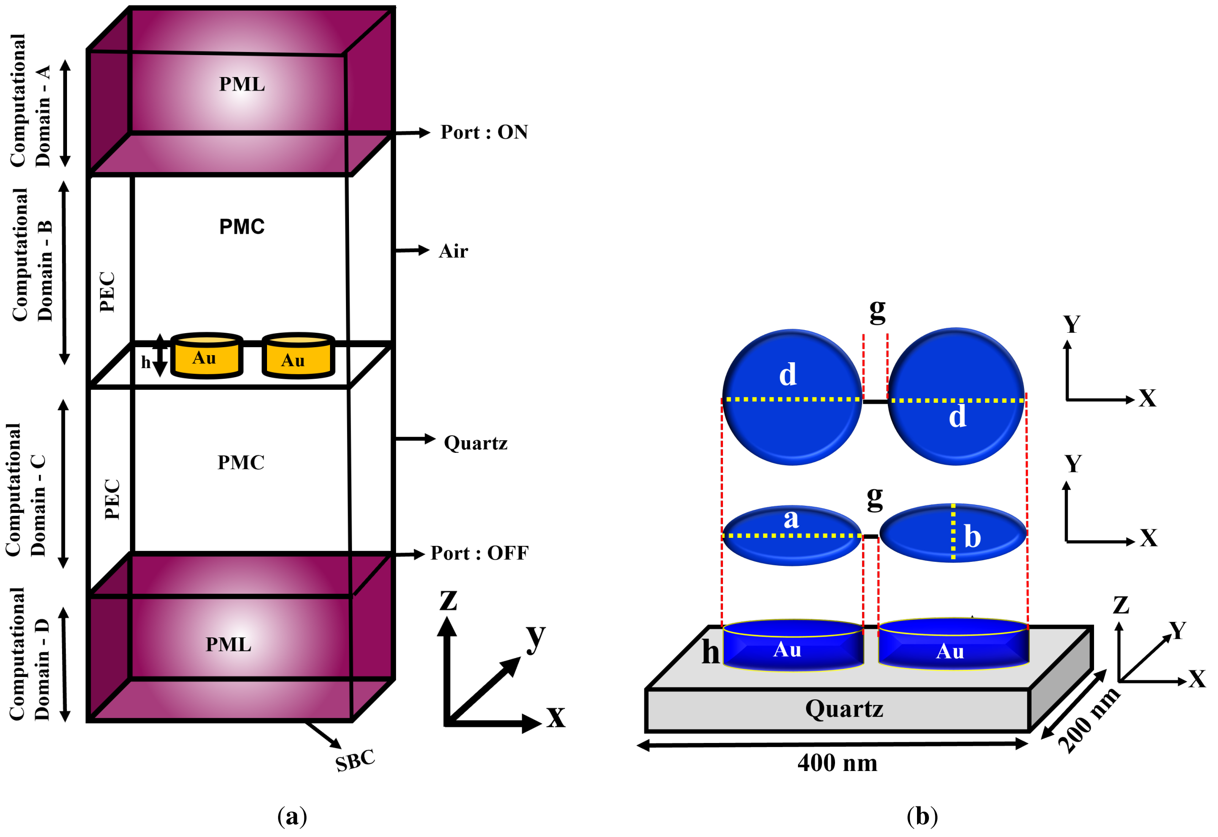

Figure 2. Designed model on COMSOL Multiphysics (a) Computational model of paired gold nano antenna along with the boundary conditions. (b) Top and Side views of a paired elliptical and circular shaped gold nano antenna.

In the FEM solver, the entire structure was discretised into 'study' mesh elemental size. Additionally, Perfectly matched layer (PML) with 200 nm height on the top of the air domain and bottom of the quartz substrate were introduced to avoid the artifacts of back reflection in the simulations. The periods along x and y directions were taken as 400 nm and 200 nm, respectively. The height of the metal disks was initially taken as 40 nm from the surface of the quartz substrate. Perfect magnetic conductor (PMC) and Perfect electric conductor (PEC) have been used along the x and y-directions, respectively to enforce the periodicity of the structure. As shown in Figure 2b, in this work unit cell has been designed to reduce the computational cost in the COMSOL but still the computational cost was quit high which cannot be neglected, hence to overcome that drawback we have developed the DL based algorithm which shows the efficient performance in terms of the computational cost. Also, it can also be said if we further reduce the COMSOL computational model to quarter structure then the computational cost can be reduced further.

ON/OFF Resonance point Description:

Next, the transmission spectra has been shown for the paired circular/elliptical structure on the quartz crystal is studied and the transmission spectra of a circular dimer with $a = b = 110$ nm, $g = 20$ nm gold nano disk and an elliptical dimer with $a = 110$ nm, $b = 10$ nm, $g = 20$ nm which were surrounded by a material of different refractive indices are shown in Figure 3. Here, it can be clearly observed that as the refractive index is increasing, the resonating wavelength is shifting towards the higher range and the

resonance dips are more sharper and stronger for elliptical shaped antenna compared to a circular structure. The sensitivity values has also been calculated 168.85 nm/RIU and 454.34 nm/RIU for circular and elliptical dimers, respectively to observe the performance of the sensing system (d = 110 nm for circular dimer, a = 110 nm, b = 10 nm and g = 20 nm for elliptical dimers).

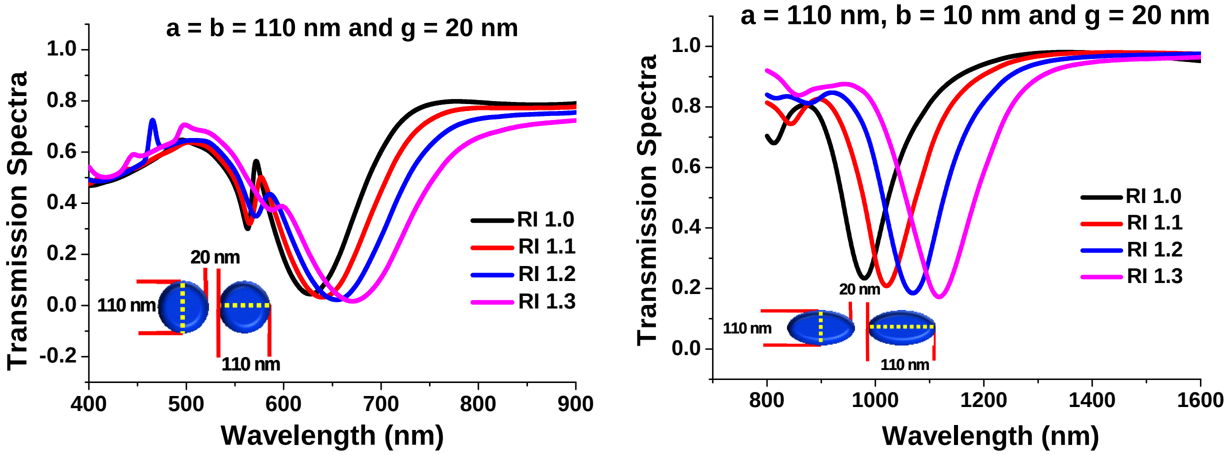

(a) (b)

Figure 3. Transmission Spectra of paired circular nano disk with $a = b = 110$ nm, $g = 20$ nm and $h = 40$ nm. (b) Transmission Spectra of paired elliptical disk with $a = 110$ nm, $b = 10$ nm, $g = 20$ nm and $h = 40$ nm.

Section II: Description of DL neural network

Training dataset discussion:

This section describes the training data that has been used to train the DL neural network. This training set helps to learn the hidden patterns and to make accurate predictions. With the help of sufficient and accurate training dataset the DL neural network shows the abilities to take the diverse inputs and weigh them with the help of corresponding activation function in the individual neurons.

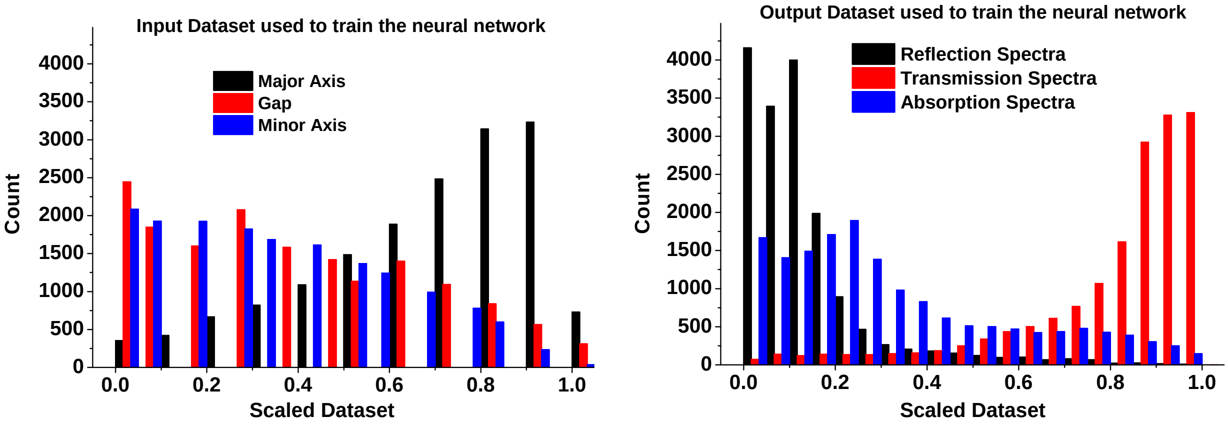

(a) (b)

Figure 4. Histogram of the Input and Output datasets for elliptical and Circular paired nano structure. (a) Shows the scaled input (Major, Minor Axis and Separation Distance) dataset used to train the neural network. (b) Shows the trend of the Output (Reflection, Transmission and Absorption Spectra) dataset used to train the developed neural network

Figure 4a shows the input training dataset that has been used to train the developed neural network where the used dataset containing 54,397 data-points (calculated from COMSOL multiphysics) for Major axis, Minor axis and Separation gap

represented by black, red and blue bars, respectively. However, Figure 4b represents the output dataset that has been used to train and to make predictions on the developed DL neural network where the used spectral dataset (calculated from COMSOL multiphysics) for reflection spectra and transmission spectra and absorption spectra are shown by black, red and blue bars, respectively.

Hyper parameter optimization to enhance the performance of DL neural network:

Typically a hyper parameter has a prominent effect on a DL neural network, hence in this section we have chosen the best set of the hyper parameters to achieve the best performance of the neural network in terms of the *MSEs* between the actual and predicted values. While making all the prediction the learning rate was set to be 0.001 in order to obtain the optimal performance. Figure 5a shows the *MSE* values when the *neurons* = 50 and *epoch* = 5000. It can be observed here that as *epoch* is increased, the*MSE* values are reduced. From this figure, it can also be seen that as the hidden layer increases the *MSE* value decreased and at *hidden layer* = 10 the calculated *MSE* are almost similar as that with *hidden layer* = 5. Hence, we have considered that *hidden layer* = 5 as the best optimized hyper parameter with almost similar *MSE* values and computational load. Figure 5b shows the calculated *MSE* when *hidden layer* = 5 and *neurons* = 50 at variable epoch. It can be seen from this this that as the epoch increases the *MSE* between the actual and predicted value decreased faster and got saturated at *epoch* = 5000. However, increment in epochs can increase the computation cost but in order to make accurate predictions with less *MSEs* we have compromised with the computational cost a bit and used *epoch* = 5000 for further observations.

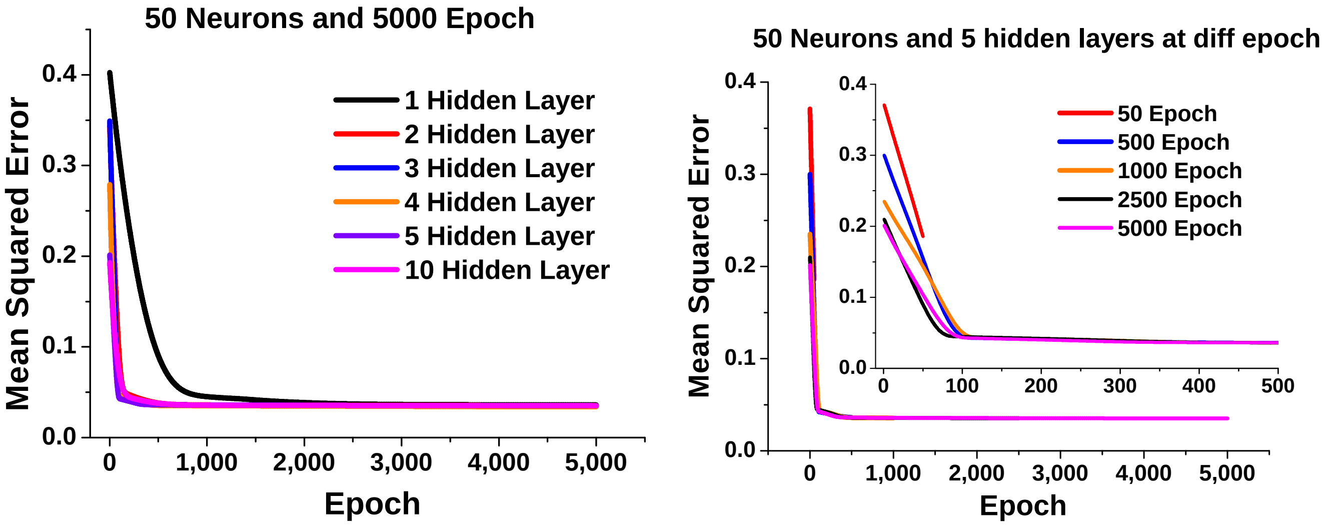

(**a**) (**b**)

Figure 5. Hyper-parameter tuning

Comparison in computational cost between the COMSOL multiphysics and the developed DL neural network:

This section discusses the comparison in the computational cost of the in-house developed DL neural network and the COMSOL multiphysics. Figure 6 shows the computational cost = 1,72,800 sec of the COMSOL multiphysics when manual meshing was used. This computational cost increases to 8,100 sec, 10,200 sec, 14,100 sec, 38,160 sec, 86,400 sec for coarse, normal, fine, finer and extremely fine meshing, respectively. However, the computational cost was significantly reduced to 236 sec when DL is used, which was an excellent agreement as in the upcoming research and modern devices computational cost plays the vitals role in order to save user's time. In this research, we offer an alternate strategy wherein we train a deep learning network to predict spectral values with reduced DL neural network processing time. The time-frames for these separate components may then be combined to determine the total execution time. This has the potential of being able to represent more complicated scenarios than linear techniques. It can also anticipate execution times for scenarios that haven't been experienced in the training data. As a result, our method may be used not only to estimate the execution time for a batch or an extended epoch, but also to aid in the selection of adequate technology and models a wide range of practical, intricate and complex photonic devices.

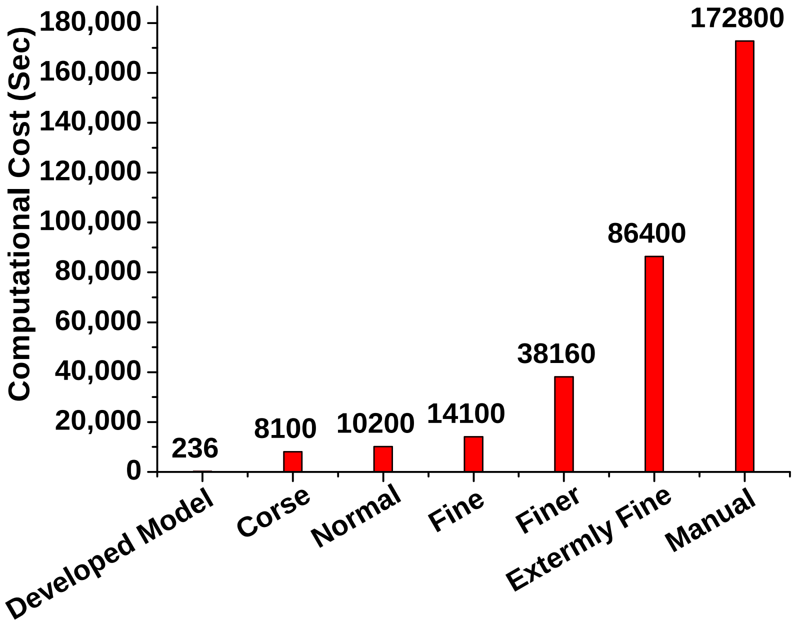

Figure 6. Comparative analysis of computational cost between Comsol multiphysics and in house developed neural network.

References

1. Johnson, P. B. & Christy, R.-W. Optical constants of the noble metals. *Physical Review B* **6**, 4370 (1972).
